# Late-Life Migration to Israel as an Ambivalent Process: Identity, Belonging, and Adaptation Among Older French Immigrants in Israel

**DOI:** 10.1007/s10823-026-09599-4

**Published:** 2026-09-12

**Authors:** Julia Adad, Ahuva Even -zohar

**Affiliations:** 1 Faculty of Social and Heath Sciences, Bar-Ilan University, Ramat Gan, Israel; 2 Faculty of Social Sciences, Ariel University, Ariel, Israel

**Keywords:** Late-life migration, Older adults, Migration to Israel, Aliyah, Identity return, Belonging, Biographical coherence

## Abstract

Migration in later life is a growing global phenomenon, raising important questions about identity, belonging, and adaptation in old age. This study explores the experiences of older adults from France who migrated to Israel (i.e. undertaking Aliyah) after the age of 65, focusing on how they make sense of this transition within the context of ageing. Using an interpretative phenomenological approach, this qualitative study draws on in-depth interviews with twelve participants in Israel (ten women and two men aged 70 and older). Rather than treating motivations, challenges, and well-being as separate domains, the analysis examines migration as a complex meaning-making process shaped by the intersection of life-course trajectories, family relationships, and cultural identity. The findings reveal an ambivalent dynamic at the core of later-life Aliyah. While migration involved substantial disruption to familiar social worlds, participants consistently interpreted it as the fulfilment of a lifelong identity project centred on family, Jewish identity, and belonging. Although they experienced persistent linguistic, social, and institutional challenges, these did not diminish their strong sense of attachment to Israel. Instead, the findings suggest that emotional belonging and everyday integration may follow different trajectories in later life, contributing to a more nuanced understanding of identity return among older immigrants. This study contributes to the literature on ageing and migration by offering a nuanced understanding of late-life mobility as a process of identity negotiation rather than simple adaptation (or its absence). It also provides insights for policymakers and practitioners supporting older immigrants, emphasizing the need to address both structural barriers and the importance of continuity, belonging, and meaningful relationships in later life.

## Introduction

Late-life migration has emerged as a growing global phenomenon, reflecting the combined effects of population ageing and increased international mobility. While migration has traditionally been associated with younger, economically active individuals, a growing body of research highlights that migration in older age involves distinct motivations, constraints, and adaptation processes (Holecki et al., 2020; Hawkins et al., 2022). In particular, older migrants often face compounded challenges related to social integration, access to healthcare, and reduced participation in labor markets, which may amplify vulnerabilities compared to younger migrant populations.

Israel provides a unique context for examining late-life migration due to its distinctive immigration regime. Under the Law of Return, Jews are granted immediate citizenship upon arrival, and a range of institutional mechanisms are designed to facilitate their integration. As a result, migration to Israel—commonly referred to as Aliyah—is not only a demographic process but also a socio-political and ideological phenomenon that differs from more conventional forms of migration (Dolberg et al., 2018).

Recent demographic trends in Israel point to relatively stable migration patterns, with Russia, the United States, and France consistently ranking among the main countries of origin (Central Bureau of Statistics, 2023). More specifically, administrative data indicate that in 2025, France accounted for approximately 15% of new immigrants (3,360 out of 22,522), ranking third after the United States (approximately 16.8%) and Russia (approximately 38.0%) (Ministry of Aliyah and Integration, 2025). At the same time, adults aged 65 and above represented approximately 12.7% of all new arrivals, underscoring the demographic significance of late-life migration within contemporary Aliyah.

Despite this notable presence, older immigrants—and particularly those from Western countries such as France—remain underrepresented in academic research. Existing studies on immigration to Israel have largely focused on populations from the former Soviet Union, Ethiopia, or the United States, often emphasizing economic integration or ethnic inequalities. In contrast, the experiences of older French immigrants, whose migration may be shaped by distinct cultural, social, and ideological factors, have received limited scholarly attention.

Addressing this gap, the present study explores the lived experiences of older French immigrants to Israel, with a particular focus on their processes of adaptation and integration. By adopting a qualitative and interpretative approach, this research aims to contribute to a more nuanced understanding of late-life migration in a unique socio-political context, while also informing policies and programs designed to support the integration of older immigrant populations.

## Theoretical Framework

### Migration in Later Life and the Challenges of Adaptation

Migration in later life has increasingly emerged as a significant field of inquiry, reflecting the intersection between global mobility and population ageing. While migration has traditionally been conceptualized as a process occurring earlier in the life course, recent research highlights that migration in older age follows distinct patterns shaped by age-related vulnerabilities, accumulated life trajectories, and structural constraints (Ciobanu et al., 2017; Fokkema & Ciobanu, 2021).

In this framework, adaptation should not be reduced to a functional adjustment to a new environment. Rather, it refers to a multidimensional process involving social integration, identity reconstruction, and the negotiation of belonging in later life (Torres, 2013; Fokkema & Naderi, 2013). Integration, in turn, encompasses not only participation in social and institutional structures but also the subjective experience of inclusion and recognition within the host society (Ciobanu et al., 2017). These processes are shaped by structural inequalities accumulated across the life course, reinforcing disparities in access to resources and opportunities in old age (Ferrer et al., 2017).

Unlike younger migrants, older adults are less embedded in labor markets and more dependent on social networks and institutional support, which affects their capacity to adapt (Ciobanu et al., 2017; Torres, 2013). Migration decisions in later life are therefore often influenced by a combination of family considerations, health needs, and lifestyle aspirations (Warnes & Williams, 2006; Fokkema & Ciobanu, 2021). However, the ability to migrate and adapt is highly selective, favoring individuals with stronger economic, social, and health resources (Warnes & Williams, 2006; Ciobanu et al., 2017).

At the same time, older migrants face significant challenges that can hinder their integration. Language barriers, in particular, play a critical role in limiting access to services, reducing autonomy, and constraining social participation (Khvorostianov et al., 2012; Torres, 2013). Research shows that these barriers are closely linked to experiences of social isolation and reduced well-being among older migrants (Dolberg et al., 2018; Koehn et al., 2020). In addition, the disruption of existing social networks and the difficulty of forming new ones in later life further complicate the adaptation process, reinforcing dependence on family or co-ethnic or co-linguistic communities (Ciobanu et al., 2017).

These challenges highlight the importance of adopting a life-course and intersectional perspective when examining late-life migration (Torres, 2013; Ferrer et al., 2017). Rather than viewing older migrants as a homogeneous group, recent scholarship emphasizes the diversity of experiences shaped by factors such as gender, socio-economic status, migration history, and cultural background (Fokkema & Ciobanu, 2021). This perspective underscores the need for qualitative approaches that capture the lived experiences and meaning-making processes of older migrants, particularly in underexplored contexts such as late-life Aliyah to Israel.

### Aliyah to Israel: A Case of Later Life Migration

Jewish migration to Israel, commonly referred to as Aliyah, represents a distinct form of migration that differs in important ways from more conventional economic or family-driven migration patterns. While most migration theories emphasize push–pull economic factors, Aliyah is deeply embedded in ideological, religious, and historical narratives that shape both the motivations for migration and the expectations associated with it (Shain, 2002). From a theoretical perspective, Aliyah can be understood as a form of ideologically driven migration, in which the act of migration is framed not only as relocation but as a return to a symbolic homeland. This notion is closely tied to Zionist ideology, which constructs Israel as both a national and spiritual center for Jewish identity. For older migrants in particular, this dimension may acquire additional meaning, as migration is often interpreted as the culmination of a life course shaped by religious education, collective memory, and long-standing attachments to Israel (Zerubavel, 1995).

Beyond its ideological and symbolic dimensions, Aliyah is also strongly structured by institutional frameworks. Israel’s Law of Return grants Jews automatic citizenship, and a range of governmental and semi-governmental organizations—such as the Ministry of Aliyah and Integration and the Jewish Agency for Israel—provide financial assistance, housing support, language programs (ulpan), and social services aimed at facilitating integration (Shuval, 1998). These institutional mechanisms distinguish Aliyah from many other forms of migration by offering structured support systems that can ease certain aspects of adaptation (Raijman & Geffen, 2018).

While these institutional mechanisms facilitate settlement and access to essential services, older migrants may nevertheless continue to face important challenges throughout the integration process. Research has shown that access to these resources does not necessarily translate into full social integration, particularly for older migrants. Structural barriers such as language acquisition, bureaucratic complexity, and mismatches between institutional services and the specific needs of older adults can limit the effectiveness of these support systems (Dolberg et al., 2018; Khvorostianov et al., 2012). Moreover, while the ideological framing of Aliyah may create strong expectations of belonging, the lived experience of migration can involve tensions between anticipated integration and everyday realities.

This tension between ideological motivation and lived experience is particularly salient in late-life migration. While Aliyah may be perceived as a meaningful or even redemptive life project, older migrants must simultaneously navigate the practical challenges of ageing in a new socio-cultural and institutional environment. This dual dynamic—between symbolic return and concrete adaptation—highlights the need to examine Aliyah not only as a national project but also as a lived, subjective experience shaped by age, identity, and social context.

### Older French Immigrants to Israel: A Specific Yet Underexplored Case

As noted earlier, immigrants from France constitute a significant proportion of recent arrivals to Israel, representing approximately 15% of new immigrants in 2025 (Ministry of Aliyah and Integration, 2025). Despite this demographic relevance, older immigrants from France remain largely underrepresented in both academic research and policy-oriented analyses. Existing scholarship on Jewish migration from France has primarily focused on broader migration trends, often emphasizing factors such as religious identity, socio-political concerns, and perceptions of insecurity in the country of origin (Anteby-Yemini & Berthomière, 2015; Ben-Rafael & Ben-Rafael, 2019). While these studies provide important insights into the motivations underlying migration, they tend to focus on younger or economically active populations, leaving the specific experiences of older migrants insufficiently explored.

Like many contemporary migrant groups, French immigrants to Israel maintain transnational ties with their country of origin. However, the French case is distinctive in that migration often involves leaving a relatively stable and affluent democratic society in which immigrants had established social, cultural, and professional lives. Aliyah may therefore be motivated less by economic necessity than by a combination of ideological attachment, Jewish identity, family proximity, and concerns about belonging and security. Continued use of the French language, consumption of French media, and reliance on French-speaking social and professional networks in Israel reflect the preservation of these pre-migration attachments rather than an absence of commitment to Israel (Amit & Bar-Lev, 2015; Anteby-Yemini & Berthomière, 2015; Ben-Rafael & Ben-Rafael, 2019).

These characteristics are particularly relevant in the context of late-life migration. For older adults, who are already more likely to depend on familiar social and cultural environments, the coexistence of strong transnational attachments and the need to adapt to a new socio-cultural context may create specific challenges (Ciobanu et al., 2017). At the same time, the ideological and symbolic dimensions of Aliyah may interact with these dynamics in complex ways, potentially reinforcing both attachment to Israel and connections to the country of origin. Despite these distinctive features, little attention has been paid to how older French immigrants experience and negotiate these multiple dimensions in their everyday lives. In particular, the intersection between ageing, migration, and identity remains insufficiently examined in this population. Addressing this gap, the present study seeks to explore how older French immigrants navigate processes of adaptation and integration, with a focus on the meanings they attribute to their migration experience.

## Methodology

### Research Design

This study adopts a qualitative research design based on Interpretative Phenomenological Analysis (IPA), an approach specifically suited to exploring how individuals make sense of significant life experiences. IPA is grounded in a phenomenological and hermeneutic epistemology, emphasizing both the detailed examination of lived experience and the interpretative role of the researcher (Smith et al., 2009; Frost, 2011). Consistent with IPA principles, the analysis follows an idiographic approach, focusing on in-depth exploration of a relatively small and purposively selected sample. The process is guided by a double hermeneutic, whereby participants seek to make sense of their migration experiences, while the researcher, in turn, interprets these meaning-making processes. This approach is particularly appropriate for examining late-life migration, as it allows for a nuanced understanding of how older adults interpret complex processes of identity, belonging, and adaptation.

### Participants and Sampling

Participants were recruited using purposive and snowball sampling strategies, in line with qualitative and IPA research practices (Smith et al., 2009). Participants were eligible for inclusion if they had migrated from France to Israel at the age of 65 or older, had been living in Israel for no more than five years, and were able to provide informed consent and participate in an in-depth interview.

The final sample included 12 participants (10 women and 2 men), aged between 70 and 92. Ten participants had adult children living in Israel, making family proximity a central feature of the migration experience for most of the sample. Five participants had purchased property in Israel prior to immigrating, while another five had maintained regular ties with the country through frequent visits for holidays or volunteer activities before their permanent relocation, suggesting that Aliyah often represented a long-term, carefully prepared life project rather than a spontaneous decision. All were able to provide informed consent and participate in an in-depth interview. The sample size is consistent with the idiographic principles of Interpretative Phenomenological Analysis (IPA), which prioritizes an in-depth understanding of individual lived experiences over statistical generalization. Accordingly, data collection was guided by the principle of analytical depth rather than data saturation, with recruitment continuing until the interviews yielded sufficient richness and convergence of themes (Smith et al., 2009; Hennink et al., 2017).

### Data Collection

Data were collected through semi-structured, in-depth interviews conducted between July 2022 and February 2023. Ten interviews were conducted face-to-face and two via video conferencing. Participants were recruited through French-speaking social media groups, community centers, and synagogues serving French-speaking communities in Jerusalem, Tel Aviv, Netanya, Ra’anana, and Modi’in, as well as through snowball sampling. All interviews were conducted in French, the participants’ native language, in order to facilitate expression and ensure the authenticity of narratives. Interviews lasted between 60 and 150 min and followed an open-ended format designed to elicit detailed personal accounts. The opening question was: “Can you tell me about your migration to Israel and your experiences since making Aliyah?” All interviews were audio-recorded with participants’ consent and transcribed verbatim in French. Given the centrality of language in IPA, particular attention was paid to the translation process. Initial analysis was conducted on the original French transcripts to preserve the meaning and nuance of participants’ accounts. Selected excerpts were later translated into English for reporting purposes. Translation was performed with attention to semantic and cultural equivalence, recognizing that meaning may not always be directly transferable across languages.

### Data Analysis

Data analysis followed the interpretative phenomenological analysis (IPA) procedures described by Smith et al. (2009). Each transcript was read and re-read several times to achieve close familiarity with the participant’s account. During the initial noting stage, descriptive, linguistic, and conceptual comments were recorded in the margins. For example, repeated expressions such as “coming home,” “finally belonging,” or “fulfilling a lifelong dream” were initially noted as reflecting participants’ symbolic attachment to Israel, whereas accounts describing reliance on French-speaking communities and difficulties communicating in Hebrew were noted as reflecting practical challenges of adaptation.

These exploratory notes were then examined for recurring patterns and grouped into broader emergent themes. For example, references to “coming home,” “finally belonging,” and achieving a lifelong aspiration were brought together under a broader theme capturing Aliyah as the fulfilment of a long-held identity project. Connections between themes were subsequently explored within each individual case and then across participants while preserving the idiographic focus of IPA. For instance, the strong sense of symbolic belonging to Israel was repeatedly found alongside participants’ continued reliance on French-speaking social networks, leading to the identification of the overarching theme of the ambivalent nature of late-life Aliyah.

Throughout the analysis, interpretation moved iteratively between participants’ accounts and the researcher’s developing understanding, reflecting the double hermeneutic that characterizes IPA, whereby the researcher seeks to make sense of how participants themselves make sense of their lived experiences. For example, participants often described Aliyah as “coming home” or “finally belonging.” Rather than taking these expressions at face value, they were interpreted alongside narratives describing continued reliance on French-speaking social networks and difficulties engaging with Israeli society, leading to a richer understanding of late-life Aliyah as an inherently ambivalent experience.

### Reflexivity and Trustworthiness

To enhance the rigor of the analysis, several strategies were employed in line with qualitative research standards (Lincoln & Guba, 1985):

- Reflexivity: A reflexive journal was maintained throughout the research process to document analytical decisions, assumptions, and the evolution of emerging interpretations. Initially, the researcher assumed that physical uprooting would constitute the primary interpretative lens through which participants understood their migration. Early analytical notes therefore organized participants' accounts into three broad categories: motivations for Aliyah, challenges of migration, and well-being. However, repeated engagement with the interviews revealed that participants did not experience these dimensions as separate or opposing. Although uprooting remained an important aspect of their narratives, participants consistently attached greater significance to symbolic belonging, ideological commitment, and the experience of "return." These reflections, documented in the reflexive journal, led to a reformulation of the analytical framework toward a more integrated interpretation emphasizing the inherently ambivalent nature of late-life Aliyah.
- Credibility: Credibility was strengthened through prolonged engagement with the data and repeated comparison between transcripts. As analysis progressed, interpretations were continually checked against participants' original narratives to ensure that the emerging themes accurately reflected their lived experiences rather than isolated quotations or the researcher's initial assumptions.
- Transparency: The analytical process was documented throughout the study, providing a clear audit trail from initial exploratory notes to emergent themes and the final thematic structure. This documentation made it possible to trace how early descriptive categories were progressively refined into higher-order interpretative themes.
- Dependability: Dependability was enhanced through the consistent application of the same analytic procedures across all interviews. Each transcript underwent the same sequence of repeated reading, exploratory noting, development of emergent themes, and cross-case comparison before being incorporated into the overall thematic framework. This systematic approach ensured that interpretations were developed consistently across all 12 cases.

The researcher occupied a position that combined both insider and outsider perspectives. Having immigrated from France to Israel at the age of 18 and lived in Israel for approximately 15 years, she shared linguistic, cultural, and migration-related experiences with participants. This facilitated rapport during the interviews and enabled a nuanced understanding of references to French and Israeli society, as well as challenges such as language acquisition and bureaucratic adaptation. At the same time, she differed substantially from participants in terms of age and life stage, having not experienced migration in later life or the health-related challenges associated with ageing. Participants were not known to the researcher prior to the study. Throughout the research process, reflexive attention was paid to how this dual insider-outsider position could both enrich interpretation and introduce assumptions, particularly when analysing participants’ narratives of belonging, adaptation, and identity.

## Results

This section presents the findings on the experiences of older French immigrants in Israel. Rather than representing discrete and independent categories, participants’ narratives reveal a complex process of meaning-making, in which migration is shaped by the interplay of ideological commitments, relational ties, and existential reflections associated with later life. Three interconnected dimensions emerge: (1) Aliyah as the culmination of a life-long project, (2) negotiating dependence and autonomy in the migration experience, and (3) navigating well-being between fulfilment and vulnerability.

### Aliyah as the Culmination of a Life-Long Project

Participants consistently described their decision to immigrate to Israel as far more than a practical response to ageing or retirement. Although individual motivations varied, their narratives reveal that Aliyah was generally experienced as the culmination of a long biographical journey rather than as a sudden life decision. Childhood memories, repeated visits to Israel, Zionist values, religious aspirations, and family relationships gradually intertwined over time, giving participants the sense that migrating to Israel represented the fulfilment of a project that had accompanied them throughout much of their lives. While these motivations can be distinguished analytically, participants rarely experienced them separately. Instead, they described them as mutually reinforcing dimensions of a single life trajectory.

### From Inherited Values to a Personal Life Project

For many participants, attachment to Israel originated within their family environment but progressively evolved into a deeply personal aspiration. Rather than simply adopting the ideals transmitted by their parents, participants described how these early influences gradually became integrated into their own identity through life experiences, repeated encounters with Israel, and an increasing sense of personal belonging. Consequently, Aliyah was not portrayed as the fulfilment of someone else’s dream, but as the realisation of a project that had progressively become their own. For example, Esti (76 years old) explains that her father transmitted a profound attachment to Israel from an early age. However, her narrative also reveals that this attachment progressively became part of her own identity:

I made Aliyah to fulfill my father’s dream. He was very Zionist and instilled in us a love for Israel from a young age… I have always loved Israel, and deep down I always knew that I would make Aliyah.

Rather than presenting Aliyah solely as an act of filial loyalty, Esti reconstructs her migration as the culmination of a conviction that had accompanied her throughout adulthood. Her father’s ideals constituted the starting point of this journey, but over time they were transformed into a deeply personal aspiration. Similarly, David (78 years old) recalls that his father’s attachment to Israel was transmitted through emotionally meaningful experiences rather than ideological discourse alone: “My father dreamed of coming to Israel. When we traveled there together in the 1970s, he turned on the car radio and said, ‘Listen, we can hear the air of Israel.” Rather than recounting this memory simply as a childhood anecdote, David presents it as one of the experiences through which Israel progressively acquired symbolic and emotional significance in his own life.

This gradual transformation of an inherited attachment into a personal project is also reflected in Batia’s narrative. Although her parents had already settled in Israel, she explains that migration had long been part of her own plans: “For me, it was obvious that I would make Aliyah after retirement… It was only my work that kept me in France.” Likewise, Myriam (75 years old) describes Aliyah as the continuation of a project that had matured over many years. Having traveled regularly to Israel to visit both her parents and, later, her daughters, she explains: “I had been travelling back and forth for several years… It had been such a long time that I had been preparing to leave.” Her account illustrates how repeated experiences in Israel gradually reinforced an existing attachment and allowed her to imagine her future there long before migration became possible.

Across participants, these narratives suggest that attachment to Israel developed progressively over the life course. Family transmission provided an initial framework, yet participants actively appropriated these values through their own experiences, relationships, and repeated encounters with the country. Rather than describing Aliyah as a decision taken in later life, they retrospectively reconstructed it as the natural outcome of a longstanding biographical process. In this sense, migration was experienced less as a new beginning than as the fulfilment of a life project that had progressively taken shape over several decades.

### Migration as Existential and Spiritual Reorientation

Beyond ideological commitment, participants describe migration as a deeply personal and existential process. Although the desire to make Aliyah had often developed over many years, significant life events prompted participants to reflect on what mattered most in the later stages of life. Migration therefore represented not only a geographical relocation but also a reorientation towards identity, meaning, and belonging. Lila (82 years old) emphasizes the intuitive and enduring nature of her attachment to Israel: “From the moment we met, my husband and I knew we wanted to live in Israel. We traveled here frequently but nowhere else.” Rather than describing a desire that emerged in old age, Lila portrays Aliyah as a long-standing aspiration shared throughout her married life. Her narrative suggests that later life did not create this project but finally allowed it to be realized, illustrating how migration was experienced as the fulfilment of a future that had long been imagined rather than as a spontaneous decision.

Similarly, Sarah (73 years old) links her migration to a moment of existential reflection following illness: “After my illness, I felt the need to reconnect with my roots. Israel brings me a sense of peace I can’t find anywhere else.” Rather than referring to geographical roots, Sarah describes a symbolic reconnection with her Jewish identity and sense of belonging. Her illness did not create this attachment to Israel; instead, it appears to have intensified an already existing desire to live in a place where she felt able to express this identity more fully. In this context, the sense of peace she associates with Israel reflects not simply physical safety but the feeling of living in accordance with values that had become increasingly important to her.

For some participants, this existential dimension is closely tied to mortality and the desire for symbolic closure. Josy (92 years old) expresses: “I was fortunate to make Aliyah at the end of my life. I feel that this is where I belong and where I should spend my final days.” Rather than expressing resignation in the face of ageing, Josy’s narrative conveys a profound sense of fulfilment. Her account suggests that making Aliyah allowed her to align the final stage of her life with a place she experienced as her true home. The feeling of belonging she describes is therefore closely linked to the perception that her personal history, identity, and future could finally be reconciled in the same place.

Taken together, these narratives suggest that later-life migration was experienced as much more than a geographical move. Rather than portraying illness, ageing, or the awareness of mortality as isolated motivations, participants described these experiences as moments that encouraged them to realize aspirations that had often existed for decades. Aliyah therefore represented an opportunity to bring together identity, personal history, and future expectations into a coherent life narrative, allowing participants to experience a stronger sense of belonging during the later stages of life.

### Relational Belonging and Intergenerational Proximity

Family relationships also played a central role in participants’ Aliyah projects. However, moving closer to children and grandchildren was rarely presented as a purely practical decision. Rather, participants described family proximity as an integral part of a long-held aspiration to build their later lives in Israel while remaining actively involved in everyday family life. Myriam (75 years old) highlights the emotional dimension of proximity: “Both of my daughters made Aliyah, and it became too hard to be apart from them.” Rather than portraying migration simply as family reunification, Myriam presents proximity to her daughters as an essential component of her own Aliyah project. Living close to them represented the opportunity to participate fully in everyday family life, transforming a long-distance relationship into a shared daily experience. Similarly, Sergio’s account suggests that what he missed most was not exceptional family events but the ordinary rhythm of everyday life. His narrative illustrates how Aliyah enabled him to share moments that had previously been experienced from afar, giving concrete expression to the family life he had long wished to reclaim:” I found it hard to miss my grandchildren’s birthdays, their graduation ceremonies, or even simple Friday night dinners.”

At the same time, these relationships involve reciprocal expectations of care. Josy (92 years old) recounts: “My daughter told me, ‘You’re too old to stay in France alone. You’re coming with us!’”. Josy’s narrative illustrates how migration decisions were also embedded in intergenerational responsibility. Her daughter’s insistence reflects a shared understanding that ageing should take place within close family relationships, making proximity an important dimension of Josy’s long-awaited move to Israel.

Conversely, Sarah’s account highlights that participants were not only seeking support from their adult children. Aliyah also enabled them to continue contributing to family life through caregiving, allowing Sarah to preserve an active and meaningful role within the family. She says: “My daughter was pregnant and insisted that I move nearby. She needed my help, and I wanted to be close to her.”

Taken together, these narratives suggest that family relationships were not experienced simply as practical reasons for migration but as integral components of participants’ lifelong Aliyah projects. Living close to children and grandchildren allowed participants to translate long-held aspirations into everyday family life in Israel. In this sense, intergenerational proximity represented not only geographical closeness but also the fulfilment of deeply held aspirations concerning belonging, continuity, and family life.

## Negotiating Dependence and Autonomy in the Migration Experience

While participants consistently described Aliyah as the fulfilment of a long-held life project, their narratives also reveal the tensions involved in adapting to a new environment in later life. These challenges extend beyond practical adjustment and are closely intertwined with questions of autonomy, identity, and social participation. Rather than portraying themselves as passive recipients of these difficulties, participants described an ongoing process of negotiating independence while adapting to everyday life in Israel.

The language barrier emerged as one of the most significant challenges shaping participants’ daily experiences. Difficulties in learning Hebrew were described not simply as cognitive or practical limitations but as obstacles to participating fully in Israeli society. Participants appeared to differ in the extent to which they had prepared for learning Hebrew before migration. However, this issue did not emerge consistently across interviews and therefore could not be explored systematically. Josie (92 years old) expresses this limitation clearly: “It was beyond my abilities. At my age, learning a new language is just too difficult. I can’t even imagine it”. Josie’s account illustrates that language learning represented more than an educational challenge. Rather, it reflected her awareness that opportunities she might once have considered attainable were now perceived as increasingly limited by age, making Hebrew acquisition difficult even to imagine. Similarly, Shlomo describes both structural and personal obstacles: *“*Ulpan was very hard for me, especially because the teacher didn’t speak French at all. I also don’t know English, so learning was tough. I must admit that I wasn’t very serious about it, so I didn’t learn much. It’s hard to go back to studying at my age. Concentrating is more difficult, and my motivation isn’t the same.”

Unlike Josie, Shlomo does not attribute these difficulties solely to ageing. His account combines limited linguistic resources, features of the learning environment, and his own reduced motivation, illustrating how participants understood language acquisition as the result of multiple interacting factors rather than age alone. Beyond the difficulty of learning, language became a marker of exclusion, as Carmel (85 years old) explains: *“*For me, the biggest difficulty is the language. I suffer a lot from not speaking Hebrew. At first, I felt like a stranger in my own world.*”* For Carmel, not speaking Hebrew extends beyond communication itself. Feeling “like a stranger in my own world” suggests that language shaped her sense of belonging and legitimacy within everyday Israeli life. Although language barriers have been widely documented in later-life migration, participants in this study often described these experiences as particularly painful because they contrasted sharply with the hopes and expectations associated with a migration that represented the fulfilment of a lifelong project.

Participants also describe difficulties in navigating institutional systems, particularly healthcare and administrative procedures. These challenges frequently reinforced feelings of dependency, not simply because of increasing age-related needs but because they limited participants’ ability to act independently within unfamiliar systems. Rachel (83 years old) highlights how language barriers affect medical interactions: *“*As long as we don’t speak the language, there are many things they don’t tell us… When the oncologist talks to me… he only stays for two minutes… I need to understand!*”*. Rachel’s frustration reflects more than communication difficulties. Her narrative illustrates the importance of participants attached to remaining active participants in decisions concerning their own health. The inability to communicate directly with healthcare professionals was therefore experienced as a loss of autonomy rather than simply an inconvenience. Similarly, Bernadette (78 years old) emphasizes the cumulative burden of everyday tasks: “At our age, we need more and more medical care, and with the language barrier and cultural differences, it’s not easy to adapt”. Rather than describing isolated administrative difficulties, Bernadette portrays these demands as gradually accumulating over time. The combination of ageing, increasing healthcare needs, language barriers, and unfamiliar institutional practices contributed to the perception that everyday tasks had become considerably more demanding following migration.

Taking together, these accounts suggest that dependency emerged through the interaction between age-related vulnerabilities and the practical realities of navigating unfamiliar linguistic and institutional environments. Participants frequently contrasted these experiences with the independence they had maintained throughout most of their adult lives, highlighting the importance they attached to preserving autonomy whenever possible.

Despite these challenges, participants actively developed strategies that enabled them to preserve autonomy while creating continuity between their lives in France and Israel. For example, Lila (82 years old) emphasizes the role of financial stability: *“*For us, the key factor was financial security… we live off our pensions…*”* Financial security appears here not simply as an economic resource but as a means of preserving independence after migration, allowing participants to establish themselves in Israel without becoming financially dependent on their children. Similarly, Miriam (75 years old) describes a gradual transition: “I traveled to Israel frequently before moving here permanently… That helped me transition more smoothly.*”* Miriam’s repeated visits suggest that adaptation began long before permanent migration. Rather than discovering an unfamiliar country upon arrival, she progressively became familiar with everyday life in Israel through repeated stays, illustrating how the future country of residence gradually became experienced as home before Aliyah itself took place.

Other strategies involved recreating familiar environments, as Lila explains: “We brought all our furniture from our home in Marseille. It made the transition much easier.” Bringing familiar furniture to Israel illustrates participants’ efforts to maintain continuity across different stages of life. Rather than leaving behind the past, they integrated familiar objects and routines into their new environment, allowing continuity between their lives before and after migration.

Overall, these narratives suggest that participants did not experience autonomy and dependency as opposing conditions but as dimensions that required continual negotiation throughout the migration process. Although Aliyah represented the fulfilment of a lifelong aspiration, preserving independence required ongoing adaptation, drawing on personal resources, familiar routines, and long-standing attachments to create a meaningful life in Israel.

### Well-Being Between Fulfilment and Vulnerability

Participants’ narratives reveal that later-life Aliyah was associated with both positive and challenging experiences, which frequently coexisted rather than representing opposite outcomes. Although participants consistently described migration as the fulfilment of a long-held aspiration, their accounts also demonstrate that well-being remained dynamic and was continually shaped by everyday experiences following migration. Many participants described a profound sense of fulfilment rooted in family relationships, social participation, and opportunities to remain actively engaged in Israeli society. Shlomo (84 years old) highlights the importance of family: “Living close to my daughter gives me a sense of security and joy.*“*. For Shlomo, family proximity represents more than geographical closeness. His narrative suggests that living near his daughter provides emotional security while reinforcing his sense of belonging during later life, allowing him to experience everyday family life in Israel rather than from a distance.

Rachel (83 years old) emphasizes the role of volunteering: *“*Volunteering gave me a new purpose. I met friends, and it became my second family. “Rachel’s account illustrates that volunteering extended beyond social participation. Through her involvement the Women’s International Zionist Organization (WIZO) and the Israeli army, she established new social relationships while continuing to contribute actively to Israeli society. These activities provided not only companionship but also a renewed sense of purpose and belonging following migration. Taken together, these accounts suggest that well-being was closely associated with maintaining meaningful social roles and relationships. Rather than simply rebuilding social networks, participants described creating new forms of participation that allowed them to remain active contributors to their communities after Aliyah.

At the same time, participants also described experiences of vulnerability, particularly when migration altered previously established identities and patterns of independence. David (85 years old), a former mathematics teacher, reflects on the loss of status: “Back home, people came to me for advice. Here, I feel limited…*”* David’s account suggests that migration involved more than leaving behind a professional career. As someone who had long occupied a respected position within his community, he experienced the loss of opportunities to share his knowledge and expertise as a disruption to an important dimension of his identity. Similarly, Carmel (85 years old) expresses feelings of dependency: “In France, I managed everything myself. Here, I need my daughter’s help for almost everything. “For Carmel, dependency was not primarily associated with declining health but with the practical consequences of navigating an unfamiliar linguistic and administrative environment. Requiring assistance with medical appointments, official procedures, and everyday administrative tasks because of the language barrier fundamentally altered her experience of independence. These narratives illustrate that vulnerability was experienced not only through practical difficulties but also through changes in identity, autonomy, and participants’ perception of themselves.

Rather than representing distinct outcomes, fulfilment and vulnerability frequently coexisted within the same migration experience. Rachel (83 years old) describes how loneliness could emerge despite her overall satisfaction: *“*In winter… I’m stuck inside. That’s when loneliness hits the hardest.*”* Rachel’s account illustrates that positive and negative experiences were not mutually exclusive. Although she described her Aliyah in highly positive terms, periods of loneliness continued to emerge under particular circumstances. Rather than contradicting her overall satisfaction, these moments highlight the evolving and dynamic nature of well-being in later life.

Overall, participants rarely portrayed Aliyah as either wholly positive or wholly negative. Instead, their narratives reveal a fundamentally ambivalent experience in which fulfilment and vulnerability continually coexist. The realization of a lifelong aspiration did not eliminate the challenges associated with ageing, language barriers, or changing social roles; rather, it reshaped the meaning participants attributed to these challenges. In this sense, well-being following later-life Aliyah emerged not as a fixed outcome but as an ongoing process of balancing belonging, purpose, autonomy, and vulnerability throughout everyday life in Israel.

## Discussion

This study aimed to explore the lived experiences of older French immigrants to Israel, with a particular focus on their motivations, adaptation processes, and well-being in later life. Using an interpretative phenomenological approach, the findings provide insight into how participants make sense of their migration within the broader context of ageing, identity, and belonging.

Rather than treating motivations, challenges, and well-being as separate dimensions, the analysis reveals a more complex and interconnected process. Across participants’ narratives, migration emerges simultaneously as a rupture from familiar social and cultural environments and as the fulfilment of a lifelong aspiration associated with personal identity, family, and belonging. Although participants encountered many of the challenges commonly described in studies of later-life migration, including language barriers, changing social roles, and increasing dependency—they rarely interpreted these experiences primarily as a story of uprooting and loss. Instead, Aliyah was consistently narrated as a symbolic return and the culmination of a long-standing personal and identity-based project. Consequently, the challenges associated with migration did not disappear; rather, they were understood through the meaning participants attributed to migration itself.

These findings suggest that the distinctive contribution of this study lies not in identifying challenges unique to later-life Aliyah, but in demonstrating how a migration experienced as a symbolic return reshapes the meaning attributed to those challenges. This perspective extends existing research on later-life migration by showing that vulnerability and fulfilment are not contradictory experiences but can coexist throughout the migration process.

The discussion therefore moves beyond a descriptive summary of the results to interpret these findings in relation to existing literature on ageing, migration, and identity, highlighting the study’s theoretical and empirical contributions.

### Later-Life Aliyah as Identity Return and Biographical Coherence

The findings suggest that identity return in later-life Aliyah should be understood less as a return to a previously lived place than as the fulfilment of a lifelong identity project through which participants achieved greater biographical coherence. Although participants experienced many of the disruptions commonly associated with migration in later life—including leaving familiar environments, adapting to a new language, renegotiating social roles, and navigating unfamiliar institutions—they rarely framed these experiences primarily as a story of uprooting. Instead, migration was understood as the continuation and fulfilment of meanings, values, and aspirations that had structured their lives over many decades.

Although previous research has frequently interpreted contemporary French Jewish migration in relation to rising antisemitism and insecurity in France (Ben-Rafael & Ben-Rafael, 2019), these factors occupied only a secondary place in most participants’ narratives. Rather than describing Aliyah primarily as an escape, participants consistently framed it as the fulfilment of a lifelong aspiration rooted in Zionist education, family transmission, religious commitment, and a profound attachment to Israel. As illustrated throughout the findings, participants repeatedly described migration as the realization of a project they had carried with them throughout their lives, allowing them to align their present lives with long-held personal and collective identities.

These findings suggest that later-life Aliyah can be understood as a process of identity consolidation, through which older adults reconnect with meanings that have structured their lives across the life course. Rather than constructing a new identity following migration, participants described Aliyah as bringing coherence between deeply held beliefs, family narratives, personal aspirations, and their everyday lives in Israel. In this sense, migration was experienced less as the beginning of a new chapter than as the completion of a life project that had remained meaningful for decades. This interpretation helps explain why participants were able to acknowledge the considerable challenges associated with migration while simultaneously describing the experience as deeply meaningful and fulfilling.

This interpretation also extends current discussions of place attachment in later-life migration. Previous research has emphasized that decisions about where to age are strongly influenced by place attachment, belonging, and life-course experiences (Sieng & Szabó, 2023). The present findings support the importance of these dimensions but suggest a different configuration in the context of later-life Aliyah. Unlike the forms of place attachment commonly described in the literature, participants’ attachment to Israel was not primarily rooted in previous residence or everyday lived experience. Instead, it had been constructed throughout the life course through Zionist education, religious traditions, family transmission, repeated visits to Israel, and a sustained emotional investment in the country. In this sense, migration did not represent a return to a place they had previously inhabited, but rather the realization of a place to which they had long felt they belonged. These findings therefore suggest that, in the context of later-life Aliyah, place attachment may be understood as a symbolic and identity-based attachment that precedes migration itself.

Taken together, these findings suggest that the specificity of later-life Aliyah does not lie in the absence of the challenges commonly associated with migration in old age. Rather, its distinctiveness lies in the way these challenges are interpreted through the experience of fulfilling a lifelong project and achieving a sense of coherence across the life course. The contribution of this study is therefore not to suggest that later-life Aliyah eliminates the disruptions associated with migration, but to demonstrate how those disruptions acquire a different meaning when migration is experienced as the culmination of a lifelong process of belonging, identity, and biographical coherence.

### Family, Meaning, and Emotionally Significant Relationships

The importance of family proximity in participants’ narratives can be interpreted through socioemotional selectivity theory, which suggests that as individuals perceive time as more limited, they increasingly priorities emotionally meaningful relationships and experiences (Carstensen, 1995; Carstensen et al., 1999). In the present study, family reunification was not merely a practical response to ageing but formed part of a broader life project centred on emotionally significant relationships and a search for meaning in later life.

Aliyah enabled participants to live closer to their children and grandchildren while simultaneously giving concrete expression to long-held Jewish, Zionist, and personal commitments. Rather than representing separate motivations, family proximity and identity-related aspirations emerged as mutually reinforcing dimensions of the same life project. This interpretation also resonates with continuity theory (Atchley, 1993), which emphasizes older adults’ efforts to preserve coherence in their identities, relationships, and personal values across major life transitions. The findings therefore suggest that later-life Aliyah should be understood as both relational and existential: a movement towards family, while simultaneously giving concrete expression to values, aspirations, and a sense of belonging that had developed throughout the life course.

### Belonging Without Complete Everyday Integration

A further finding of this study is that participants distinguished between their profound sense of belonging to Israel and the practical realities of everyday life following migration. Although they consistently described Aliyah as the fulfilment of a deeply meaningful personal, religious, and identity-based project, this did not necessarily translate into complete participation in every aspect of everyday Israeli society.

Participants frequently described persistent language barriers, difficulties navigating administrative and healthcare systems, reliance on family members for everyday interactions with institutions, and the continued importance of French-speaking social networks. As illustrated in the findings, these strategies were not described as expressions of weak commitment to Israeli society but as practical adaptations that enabled participants to manage everyday life despite the challenges associated with migration in later life. These findings suggest that a strong sense of national and identity-based belonging does not necessarily imply complete practical integration. Participants often expressed a profound attachment to Israel while simultaneously relying on familiar linguistic, familial, and community resources in their daily lives. Emotional belonging and practical integration therefore appeared to follow different trajectories rather than developing simultaneously.

This interpretation is consistent with previous research showing that older migrants frequently rely on co-ethnic networks, familiar languages, and community resources as adaptive strategies during migration (Horn & Schweppe, 2016). The present findings support these observations but further suggest that, in the context of later-life Aliyah, these adaptive strategies coexist with a particularly strong sense of identification with Israeli society. Rather than reflecting limited commitment or divided loyalties, reliance on French-speaking environments represented a practical response to ageing and migration while participants simultaneously expressed a profound sense of belonging to Israel.

Taken together, these findings indicate that the distinctive contribution of this study does not lie in demonstrating forms of adaptation unique to later-life Aliyah. Instead, it highlights how practical dependence on familiar linguistic and social resources can coexist with a strong sense of national, cultural, and identity-based belonging. This distinction helps explain why participants could experience themselves as fully belonging to Israel while continuing to encounter significant practical barriers in their everyday lives.

### Reconstructing Home and Well-Being in Later-Life Aliyah

The findings also provide new insight into how older adults reconstruct a sense of home and negotiate well-being following migration. Although later-life migration is often associated with disruption, participants described actively recreating familiarity in Israel by bringing personal belongings, maintaining French cultural practices, preserving everyday routines, and relying on French-speaking social networks. These practices did not eliminate the challenges associated with migration but helped participants establish continuity in their everyday lives while adapting to a new environment.

Rather than leaving one home behind and creating an entirely new one, participants described carrying meaningful elements of home across national borders. In this sense, home emerged less as a fixed geographical location than as a set of relationships, routines, cultural practices, memories, and personal meanings that could be reconstructed in a different setting. This interpretation is consistent with recent work suggesting that ageing in place should be understood as a dynamic process rather than remaining permanently attached to one physical location (Lewis & Buffel, 2020). Likewise, studies of older migrants have shown that familiarity with one’s living environment, housing, and everyday routines contributes significantly to subjective well-being following migration (Phlix et al., 2023).

The present findings extend these perspectives by suggesting that the reconstruction of home following later-life Aliyah was facilitated by participants’ longstanding symbolic attachment to Israel. As demonstrated throughout the findings, Israel occupied an important place in participants’ family histories, Zionist education, religious beliefs, repeated visits, and future aspirations long before migration occurred. Consequently, although Israel represented a new place of residence, it was not experienced as entirely unfamiliar. Rather than beginning from a blank slate, participants progressively transformed a place that had long held symbolic significance into an everyday lived home.

Participants’ narratives also illustrate that well-being after migration remained fundamentally dynamic. Consistent with previous research, participants experienced both important resources and significant vulnerabilities, including family support, meaningful social engagement, language barriers, dependence on relatives, loneliness, and changes in social status (Ciobanu et al., 2017; Fokkema & Ciobanu, 2021; Hawkins et al., 2022). These findings confirm that fulfilment and vulnerability frequently coexist in later-life migration and should not be understood as mutually exclusive outcomes. However, the contribution of the present study does not lie in suggesting that this coexistence is unique to later-life Aliyah. Rather, it lies in demonstrating how participants interpreted these vulnerabilities through the meaning they attributed to migration itself. Throughout the interviews, practical challenges did not negate participants’ sense of fulfilment because migration was consistently understood as the culmination of a lifelong project rooted in identity, family, religious commitment, and belonging. Consequently, difficulties such as language barriers, administrative dependence, or loneliness became integrated into a broader life narrative rather than being experienced solely as signs of unsuccessful adaptation.

Taken together, the findings suggest that the specificity of later-life Aliyah lies less in the objective experiences of migration than in the subjective meaning participants attributed to those experiences. While many of the practical challenges described in this study are shared by older migrants in diverse contexts, participants consistently interpreted them within a narrative of fulfilment rather than one of loss. Later-life Aliyah was therefore experienced not as the interruption of a life trajectory, but as its completion. In this sense, the principal contribution of this study is to show how migration in later life can foster biographical coherence, enabling older adults to integrate both fulfilment and vulnerability into a meaningful life story centred on belonging, family, and the realisation of a lifelong aspiration.

### Implications for Policy and Practice

The findings of this study have several implications for policies and services supporting older immigrants. Although many participants expressed a strong sense of belonging to Israel, they also described persistent practical challenges related to language, healthcare navigation, administrative procedures, and everyday participation. These findings suggest that successful integration in later life requires attention not only to immigration itself but also to the specific needs associated with ageing.

First, Hebrew language programmes could be better adapted to older learners. Participants frequently described limited Hebrew proficiency as one of the main barriers to independent participation in everyday life. Age-sensitive language programmes that progress at a slower pace, incorporate French-language support when appropriate, and are embedded within social or cultural activities may better meet the needs of older immigrants than models primarily designed for labour-market integration.

Second, French-speaking community networks should be recognised as valuable resources within the integration process. Rather than replacing broader participation in Israeli society, these networks provide continuity, emotional support, practical assistance, and opportunities for social participation while individuals adapt to a new environment. Policies could therefore build on these existing community resources by promoting bilingual activities, intergenerational initiatives, and partnerships between francophone organisations and mainstream community services.

Third, healthcare and administrative services should become more accessible to older immigrants. Participants frequently described difficulties understanding information, navigating complex procedures, and communicating with professionals. Providing bilingual information, navigation support, and culturally responsive services may improve access to care and reduce dependence on family members, particularly for older adults living with chronic illness or functional limitations.

More broadly, the findings suggest that policies supporting older immigrants should adopt a life-course perspective that recognises the intersection of ageing and migration. Practical assistance should not be viewed solely as facilitating initial settlement but as supporting long-term participation, autonomy, and well-being throughout later life.

### Limitations and Future Research Directions

This study should be interpreted in light of several considerations. The sample was predominantly composed of women (ten women and two men). Although no systematic gender differences emerged within the interviews, men’s experiences of later-life Aliyah remain less extensively represented and warrant further exploration.

Participants were recruited primarily through community organisations, synagogues, social media, and snowball sampling. Consequently, the study may have captured the experiences of individuals who were relatively connected to francophone community networks. Future research could include older immigrants who are more socially isolated or less engaged with organised community life in order to explore a wider range of adaptation experiences.

Finally, this study offers a cross-sectional perspective on later-life Aliyah. Longitudinal research would provide valuable insight into how belonging, adaptation, identity, and well-being develop throughout the migration process and across different stages of later life. Future studies could also compare older immigrants from France with older immigrants from other countries of origin, including the former Soviet Union, Ethiopia, North America, and other Western countries. Such comparative work would help distinguish experiences that are common to later-life migration from those that are more closely related to the particular historical, cultural, and symbolic context of Aliyah. In addition, research examining return migration or circular mobility between France and Israel would further enrich understanding of mobility, belonging, and ageing among older French immigrants.

## Conclusion

This study demonstrates that later-life Aliyah among older French immigrants is best understood as a process through which participants integrated the challenges of migration into a coherent life narrative. Although migration involved substantial disruptions—including changes in language, social relationships, everyday routines, and social roles—participants consistently interpreted these experiences as the fulfilment of a lifelong project shaped by family, Jewish identity, belonging, and personal meaning. Rather than experiencing migration primarily as a rupture, they understood it as the completion of an important chapter of their life course. The findings also show that a strong sense of belonging to Israel did not necessarily translate into complete everyday integration. Participants frequently relied on family members, French-speaking social networks, and familiar cultural practices while continuing to face linguistic, administrative, and institutional challenges. These findings suggest that emotional belonging and practical integration may follow different trajectories in later life.

The principal contribution of this study is to offer a more nuanced understanding of identity return in later-life Aliyah. Rather than representing a geographical return, participants experienced identity return as the fulfilment of a lifelong project that gave coherence to their life trajectory despite the challenges associated with migration in old age. In this sense, the concept of biographical coherence helps explain why fulfilment and vulnerability could coexist throughout participants’ narratives. By highlighting the distinction between subjective belonging and everyday participation, this study contributes to a more nuanced understanding of migration, ageing, and identity in later life, while underscoring the importance of policies that recognize the intersecting realities of ageing and migration.

## Data Availability

The qualitative data (interview transcripts) that support the findings of this study are not publicly available due to privacy and ethical restrictions, in accordance with the approval of the university’s ethics committee. Summary information may be provided by the corresponding author upon reasonable request.
